# Spectral deep learning‐based patient and bowtie scatter correction for clinical photon‐counting CT

**DOI:** 10.1002/mp.70442

**Published:** 2026-04-28

**Authors:** Lukas Hennemann, Julien Erath, Andreas Heinkele, Eric Fournié, Martin Petersilka, Karl Stierstorfer, Marc Kachelrieß

**Affiliations:** ^1^ Division of X‐ray Imaging and CT German Cancer Research Center (DKFZ) Heidelberg Germany; ^2^ Computed Tomography Division Siemens Healthineers AG Forchheim Germany; ^3^ Medical Faculty Heidelberg Heidelberg University Heidelberg Germany

**Keywords:** bowtie, photon‐counting CT, scatter correction

## Abstract

**Background:**

The presence of scatter in computed tomography degrades image quality, and can be caused by the patient and by other components in the beam path, such as the bowtie filter. While conventional energy‐integrating detectors do not provide spectral distinction, photon‐counting (PC) detectors are energy‐selective and provide spectral information about the incoming X‐ray photons. Since each energy threshold is affected differently by scatter, this spectral information implicitly encodes the scatter content of a projection.

**Purpose:**

The purpose of this work is to investigate how the spectral information can be exploited to improve deep learning (DL)‐based scatter correction. Furthermore, the performance of joint and separate patient and bowtie scatter correction will be investigated, addressing that bowtie scatter has not been considered in current DL‐based approaches.

**Methods:**

We present a DL‐based approach that can estimate bowtie and patient scatter jointly and compare it against a separate correction. We also introduce neural network‐based methods that incorporate the spectral information inherent in PCCT for scatter correction. We present networks that estimate scatter for up to four energy thresholds simultaneously. Training and validation was performed with Monte Carlo data as well as with real data measured by a clinical PCCT system.

**Results:**

When comparing joint and separate patient and bowtie scatter estimation, both methods reduce the mean absolute error (MAE) from 8 HU to 1 HU. All proposed DSE methods effectively reduce scatter artifacts and perform better than the convolution‐based reference approach. Incorporating the spectral information further improves the performance, with the DSE variant with four energy thresholds achieving the best overall results for all thresholds. For all energy thresholds tested, the spectral DSE methods reduced scatter errors originating from the patient and the bowtie in PCCT from up to 8 HU to below 1 HU. In addition to the global MAE, we report a critical MAE (MAE_10_) restricted to voxels with uncorrected errors > 10 HU, as such deviations are visually perceptible in soft tissue and exceed the noise level of modern CT systems. In all test cases, the proposed spectral methods reduced the MAE_10_ from 23.8 HU in the uncorrected images to 1.6 HU after spectral correction. The affected voxels comprised on average 25 % of the image volume, indicating a significant reduction in artifact intensity in the most affected areas. In virtual monoenergetic images (VMI), the application of spectral neural networks resulted in a significant reduction in MAE from ∼16 HU to ∼2 HU at 45 keV, from ∼8 HU to ∼1 HU at 70 keV, and from 5 HU to under 1 HU at 100 keV.

**Conclusions:**

This paper presents a combined method for correcting patient and bowtie scatter that delivers results equivalent to separate corrections and thus eliminates the need for multiple networks. Further, we demonstrate that deep scatter estimation can effectively exploit the spectral information available to improve scatter correction, especially for spectral applications, like VMIs. Spectral DSE networks slightly outperformed non‐spectral variants, with multiple energy thresholds lead to more accurate estimations. This enables the use of one network for scatter correction and eliminates the need for multiple ones, thereby saving computational cost and complexity.

## 1 INTRODUCTION

**FIGURE 1:**
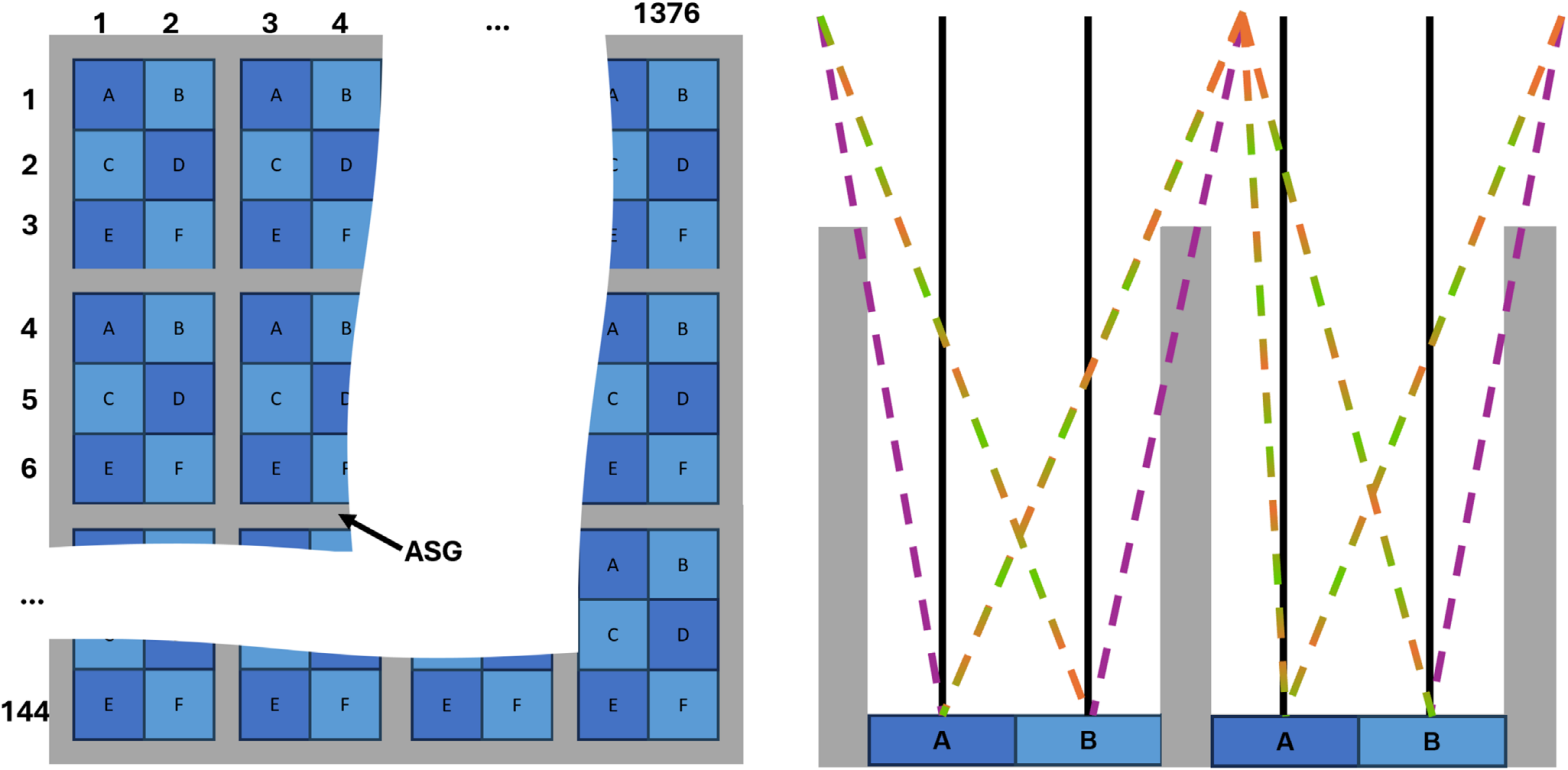
Left: Simulated detector geometry with six pixels (Std mode) between the lamellae of the ASG. Right: Attenuation of the incoming photons depending on their incident angle. The black lines visualize the primary intensity. The orange‐green lines show non‐attenuated scattered photons, whereas purple lines represent photons attenuated by the ASG. The pixel position‐dependent attenuation explains the high‐frequency characteristics of the scatter distribution.

**FIGURE 2:**
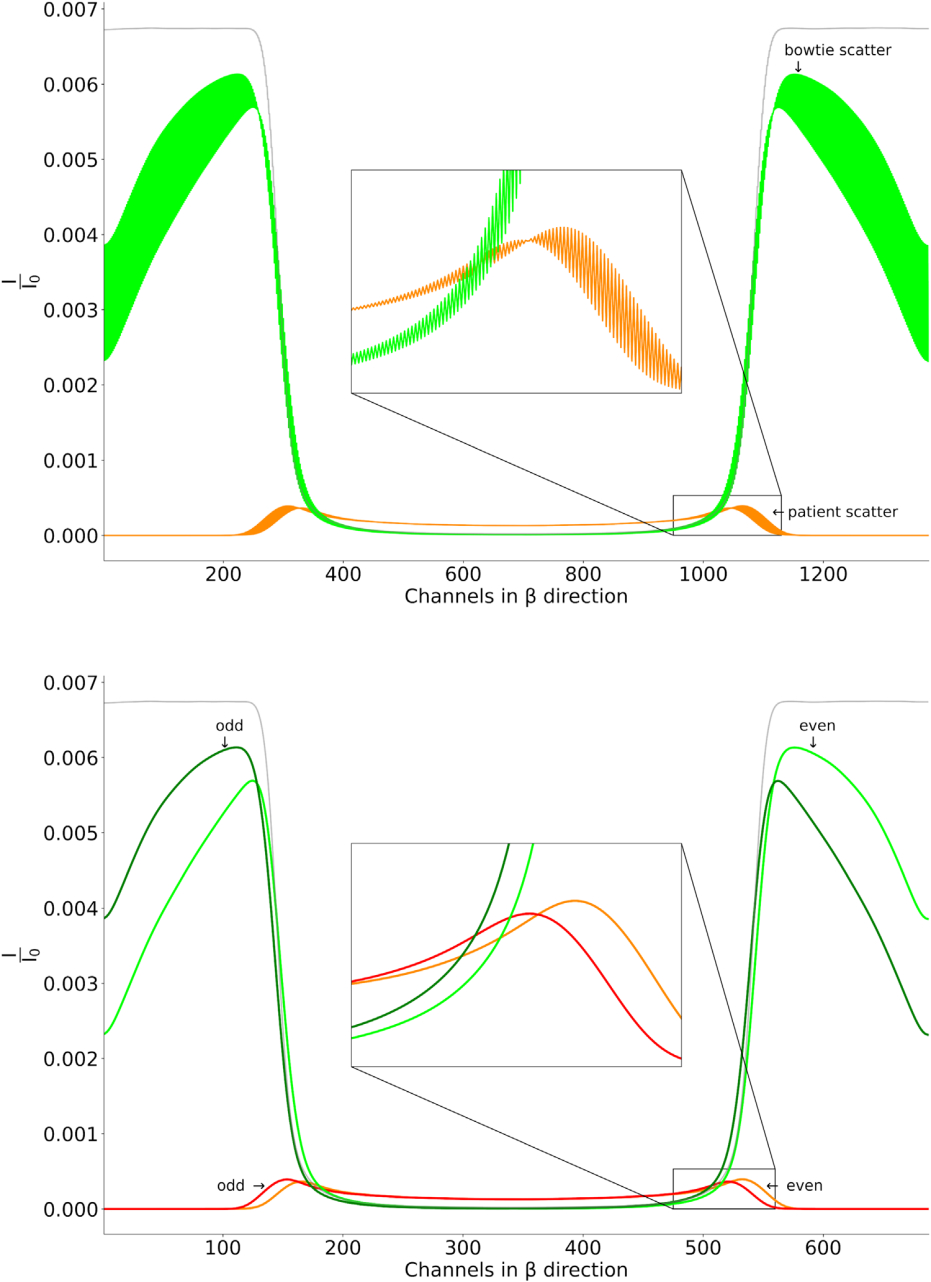
Top: Scatter distribution obtained from MC simulations, showing contributions from both bowtie (green) and patient (orange) scatter of a 30 cm water phantom. The distribution is averaged across all detector rows. The zoomed‐in region highlights high‐frequency components in the scatter signal, attributed to the coarse ASG. Bottom: The scatter signal is further separated into contributions from the odd and even pixels within the ASG, revealing its low‐frequency characteristics. The light green and orange lines indicate even channels, while dark green and red represent odd channels. The gray line is the primary intensity of the water phantom, scaled to fit the diagram.

Photon‐counting computed tomography (PCCT) was introduced in clinical routine in 2021.^1^, ^2^, ^3^ In conventional energy‐integrating (EI) detectors, scintillators (e.g., gadolinium oxysulphide) are used to detect the incident X‐rays. The absorption results in the emission of optical photons, which in turn are converted into an electrical signal by photodiodes. This indirect detection process of X‐ray photons is relatively slow and prevents the counting of individual photons and the retention of their energy information.^4^ In contrast, in photon‐counting (PC) detectors the sensor material is a semiconductor (e.g., cadmium telluride, CdTe). These semiconductors are used to generate a charge cloud when an X‐ray photon arrives and interacts with the sensor. This charge cloud can be measured directly as an electrical signal. This direct conversion allows individual photons to be counted and makes PC detectors energy‐selective, that is, they provide spectral information about the energies of the X‐ray photons interacting with the detector.^1^, ^5^, ^6^ This capability unlocks a wide range of diagnostic applications, including multi‐material decomposition, virtual monoenergetic imaging and K‐edge imaging, while simultaneously offering reduced radiation dose, improved spatial resolution and enhanced image contrast.^7^, ^8^, ^9^, ^10^ However, PCCT remains subject to fundamental limitations of X‐ray imaging physics, among them scatter. Scattered radiation originates from patient or surrounding hardware, such as bowtie or other filters. The scattered photons that are not absorbed by the anti‐scatter grid (ASG) contribute to the detector reading and lead to image degradation due to artifacts such as cupping, streaking, or ring‐like features. Moreover, scatter distorts the spectral distribution of detected scattered photons. These effects interfere for example with accurate material decomposition and are particularly disruptive at low photon energies, which are most sensitive to scatter but essential for spectral imaging.^11^, ^12^, ^13^ To minimize scatter, CT systems employ ASGs. In PCCT however, the detector pixel size is reduced to mitigate issues like pulse pile‐up. This miniaturization limits the effectiveness of fine‐pitch grid designs, necessitating the use of coarser ASGs. These coarse grids where several — for a typical clinical PCCT system: six in standard (Std) and 24 in ultra high resolution (UHR) scanning mode — pixels are surrounded by the ASG (see Figure 1), in turn may introduce secondary artifacts, such as ring artifacts, due to spatial aliasing of the ASG structure itself. As a consequence, the typical low frequency scatter is now visible in high frequency domain since each pixel perceives a different portion of the scatter depending on its relative position within the ASG. The angle of incidence of the scattered photons is responsible for whether a photon is attenuated by the ASG on its way to the detector or measured directly by the detector. Figure 2 shows such a high frequent scatter distribution which is caused by the coarse ASG.

**FIGURE 3:**
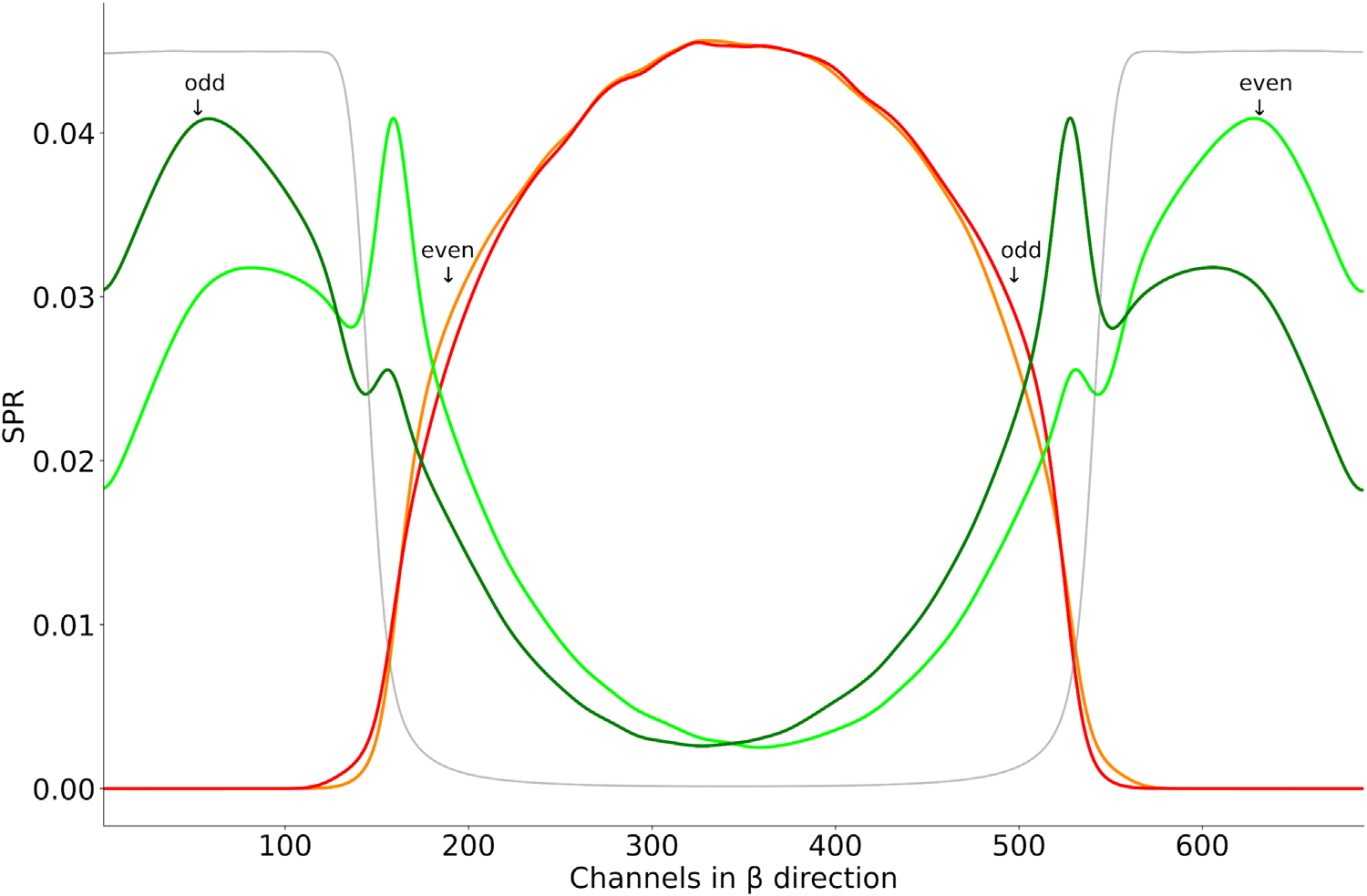
Scatter‐to‐primary ratio derived from MC simulations of a 30 cm water phantom. Both bowtie (green/light green) and patient (red/orange) SPRs are shown, averaged across all detector rows. The signal is decomposed into contributions from odd and even detector pixels within the ASG, illustrating the low‐frequency nature of the scatter distribution. Light green and orange lines correspond to even channels, while dark green and red represent odd channels. The gray curve shows the primary intensity of the water phantom, scaled for visual comparison. Notably, at the object edges, the bowtie SPR, and thus the bowtie‐induced scatter, dominates over the patient SPR.

Given these developments, there is a need for robust and spectrally aware scatter correction methods that preserve the quantitative accuracy of PCCT while maximizing its spectral benefits. The aim of this work is to investigate and develop advanced correction strategies for clinical PCCT, focusing on the integration of spectral information into data‐driven models, and to evaluate their impact on image quality, artifact suppression, and quantitative accuracy. Accurate scatter correction in PCCT requires dedicated spectral methods to preserve the energy‐resolved information critical for material decomposition and quantitative imaging. Among the model‐based approaches, Lewis et al.^14^ proposed an energy‐bin‐specific scatter estimation and correction framework that significantly improves iodine quantification by addressing the energy dependence of scatter in spectral imaging systems. On the other hand, several convolutional neural network (CNN)‐based methods for scatter correction in non‐spectral CT and CBCT contexts have been developed. These include U‐shaped neural networks in the projection domain and residual networks, which are able to reduce forward scatter artifacts. Among these, the deep scatter estimation (DSE) frameworks, the first methods of this kind, have emerged as particularly effective and robust solutions for clinical CT systems.^15^, ^16^ DSE networks enable fast inference‐time estimation of scatter and have been successfully extended to address cross‐scatter correction.^17^ Recent advancements have further demonstrated that DSE‐based scatter estimation can be performed without the need for computationally expensive Monte Carlo (MC) simulations.^18^ Moreover, the application of DSE on fourth‐generation CT scanners has yielded promising results in mitigating scatter.^19^ While these methods effectively correct scatter artifacts introduced by the scanned patient, they do not correct for scatter originating from other objects in the beam path, such as prefilters or bowtie filters for example. The latter are primarily used to modulate the X‐ray intensity depending on the beam position, with the purpose of optimizing dose distribution and enhance image quality.^20^, ^21^, ^22^, ^23^ The bowtie filter has an inhomogeneous geometry along the fan (β) direction, resulting in position‐dependent attenuation and, consequently, a varying scatter‐to‐primary ratio (SPR)(see Figure 3). This ratio directly correlates with errors in the reconstructed image and contributes to artifacts.^17^ In addition, the bowtie filter is the last filter before the patient and therefore contributes most to the scatter that irradiates the patient. This work therefore only deals with the bowtie filter, which is representative of all other prefilters. Furthermore, all previous mentioned DSE and other PC scatter correction methods do not incorporate the available spectral characteristics of the PC data. This highlights a current gap in research, which could potentially further improve correction accuracy and image quality. Motivated by these findings, we present a novel framework for spectral scatter correction in PCCT. Building on prior work of Hennemann et al.,^24^ who introduced the first DSE architecture capable of processing spectral data and explored initial applications for bowtie‐induced scatter. Our here presented work extends this line of research by introducing four new different network structures that incorporate the spectral information of the incoming photons for scatter correction. To improve the performance of scatter correction, we present network architectures that are able to estimate scatter for up to four energy thresholds simultaneously, taking into account the spectral information of the photons. The SPR in PCCT is known to vary across energy thresholds, with the lowest threshold yielding the highest SPR and vice versa.^25^, ^26^, ^27^ We leverage this spectral dependency of scatter by incorporating multiple energy thresholds as input to the proposed neural networks. Our networks are also designed to take into account the coarse structure of the ASGs. For this purpose, the entire detector signal is divided into several sub‐signals, whereby each sub‐signal is assigned to pixels that have the same relative position to the ASG. In addition, a detailed consideration is made as to whether bowtie and patient scatter should be estimated jointly or separately by DSE. Furthermore, a detailed evaluation of the spectral and non‐spectral DSE architectures is carried out with regard to the number of energy thresholds used for the estimation and correction. We also demonstrate the application of the proposed networks to measurement data from a clinical PCCT system. The organization of the paper is as follows: Section 2 describes the details about our MC generated data set, the proposed network architectures, the reference method, as well as the training and evaluation details. In Section 3 the network results are shown and compared in projection and in image domain. Finally, we discuss our findings and provide a short outlook.

## 2 MATERIAL AND METHODS

### 2.1 Simulated training data set generation

**FIGURE A1:**
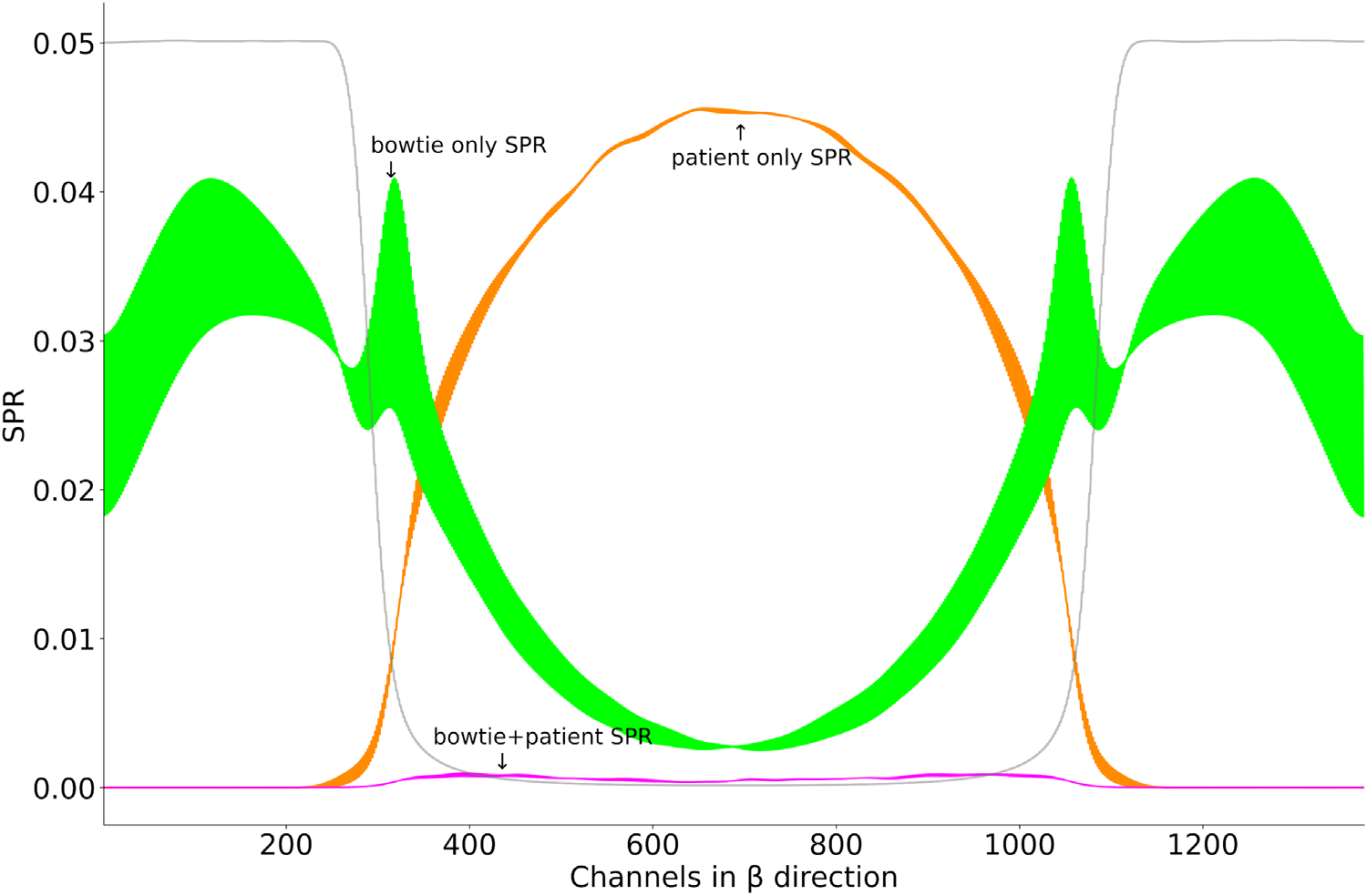
Scatter‐to‐primary ratios derived from MC simulations of a 30 cm water phantom. The bowtie (green), the patient (orange) and also the SPR from photons scattered in the bowtie as well as in the patient (pink) are shown. The gray curve shows the primary intensity of the water phantom, scaled for visual comparison.

The simulated data used in this work were generated using the MOCASSIM (Siemens Healthineers AG, Forchheim, Germany) MC simulation software.^28^, ^29^ The simulation was adapted to match the geometry of the PCCT scanner NAEOTOM Alpha.Peak^®^ (Siemens Healthineers AG, Forchheim, Germany). A detector size of 1376 × 144 pixels was simulated. In addition, a 2D ASG was taken into account which always comprises of 2 × 3 pixels in standard (Std) mode. The system is also capable to scan in UHR mode which enables detector dimensions up to 2752 × 288 pixels, but this work solely focus on the Std mode. The exact specifications and parameters of the simulation can be found in Table 1. Figure 1 shows the simulated detector geometry with six pixels between the lamellae of the coarse ASG. However, the remaining parameters such as grid height, thickness and width are proprietary and cannot be disclosed. We confirm that the configuration used in this study is identical to that of the real PCCT system employed for the measurements and test data, ensuring that the reported results accurately reflect the performance of the actual hardware. The proposed methods are validated assuming four energy thresholds and can be extended to any number of energy thresholds. The following four energy thresholds were simulated at 20, 55, 70, and 90 keV. These represent a setting which is also available at the scanner in research mode. The polychromatic simulation was performed with 140 kV tube voltage and $10^{9}$ simulated photons per projection. This specific tube voltage is used because it is a possible voltage value in the quantum plus mode, which utilizes the energy information. In addition to the scatter component of the patient, that is, photons that are only scattered in the patient, the bowtie scatter (only from the standard bowtie filter) was also simulated (our MC simulation also accounts for scatter of second or higher order). Figure A1 in Section A.1 shows the SPR for a 30 cm water phantom, broken down into contributions from bowtie scatter, object scatter, and photons that scatter in both the bowtie and the patient filter. Including this scatter component increases the MAE of the full test dataset by only about 0.4 % (under 0.1 HU), while the SPR magnitude is roughly two orders of magnitude smaller compared to the other scatter types. Moreover, in the context of evaluating whether bowtie‐ and patient‐related scatter can be estimated jointly, this scatter type would have been difficult to classify consistently. For these reasons, it was omitted. We simulated different water and semi‐anthropomorphic phantoms of different sizes and positions. Simple elliptical and cylindrical water phantoms were simulated with sizes between 20 and 40 cm diameter (15 phantoms each). For more complex training data, the FORBILD^30^ thorax (60 phantoms) was used. We introduced a scaling factor between 0.7 and 1.3 which modifies the standard phantom definition. A random shift between −8 and 8 cm in y‐direction is performed for each phantom size. The MC simulations of the phantoms were carried out in 5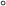 steps, resulting in a total data set of 7560 data pairs of scatter for each energy threshold. We performed a 80:20 split for training and validation. For the testing data set we simulated additional 45 head FORBILD phantoms, which were not used at all during training and validation. Further testing was carried out with simulated patient data. Therefore different XCAT^31^, ^32^ phantoms of average and obese persons were simulated at different z positions with the same previously mentioned simulation parameters. The simulation was based on a mesh implementation of Dettmer et al.^33^ to save simulation time.

**TABLE 1:** Simulation setup: (A) CT system specifications and (B) Monte Carlo parameters.

A) Technical specifications of the simulated CT system	
Number of detector pixels in β direction	1376
Number of detector pixels in z direction	144
Field of measurement diameter	50 cm
Detector shape	cylindrical
Source to detector distance	1113 mm
Source to isocenter distance	610 mm
Pixel size at the isocenter (UHR mode)^6^	0.151×0.176 mm^2^
Pixel size at the isocenter (Std mode)^6^	0.302×0.352 mm^2^
**B) Monte Carlo parameters and characteristics**	
Code & version	MOCASSIM (Siemens Healthineers), v10.41
Validation	Stierstorfer et al.^28^, ^29^
Source model	Point source at the focal spot; emission restricted to detector acceptance; beam filtration modeled via filtered spectra
Cross‐sections	EADL and EPDL (NEA/LLNL libraries)
Transport parameters	Photon‐only; Compton/Rayleigh from differential cross‐sections; isotropic K‐fluorescence; termination at geometry exit/trap; collimators and grids included
Scored quantities	Photon classes: primary, patient scatter, bowtie scatter, combined scatter
Variance reduction	None
# histories	$10^{9}$ photons per projection

**FIGURE 4:**
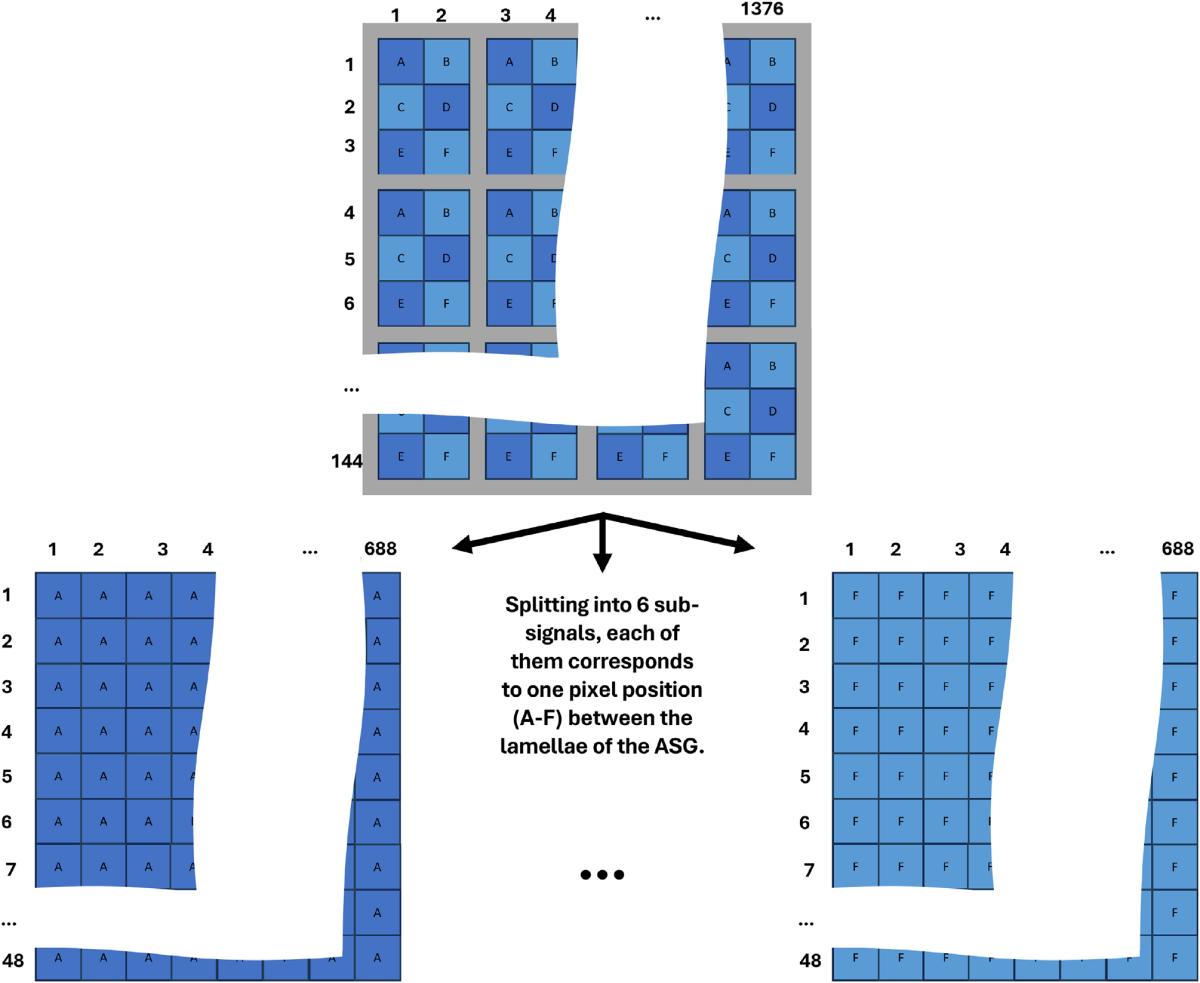
The detector signal is divided into six different sub‐signals, each corresponding to a different pixel position (A‐F) within the lamellae of the ASG. These six signals are then processed by the neural network. The merging takes place in reverse, whereby the entire detector signal is obtained by alternating the sub‐signals according to their position within the ASG.

**FIGURE 5:**
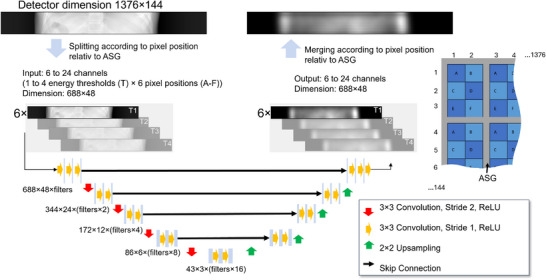
The neural network receives at least six different network inputs so that the different pixel positions (A‐F) between the lamellae of the ASG can be taken into account. It is trained in such a way that at least six output channels are estimated, which are then merged to form a complete detector signal. Depending on the network, a multiple of the six input or output channels are used. For clarity, the six input and output channels are not listed individually in the image, but are abbreviated with the “6 ×” symbol. A more detailed overview of the number of used in‐ and outputs is given in Table 3. If more than six in‐ or output channels are used, this is referred to as a spectral network. The additional channels are highlighted in gray in this diagram.

**FIGURE 6:**
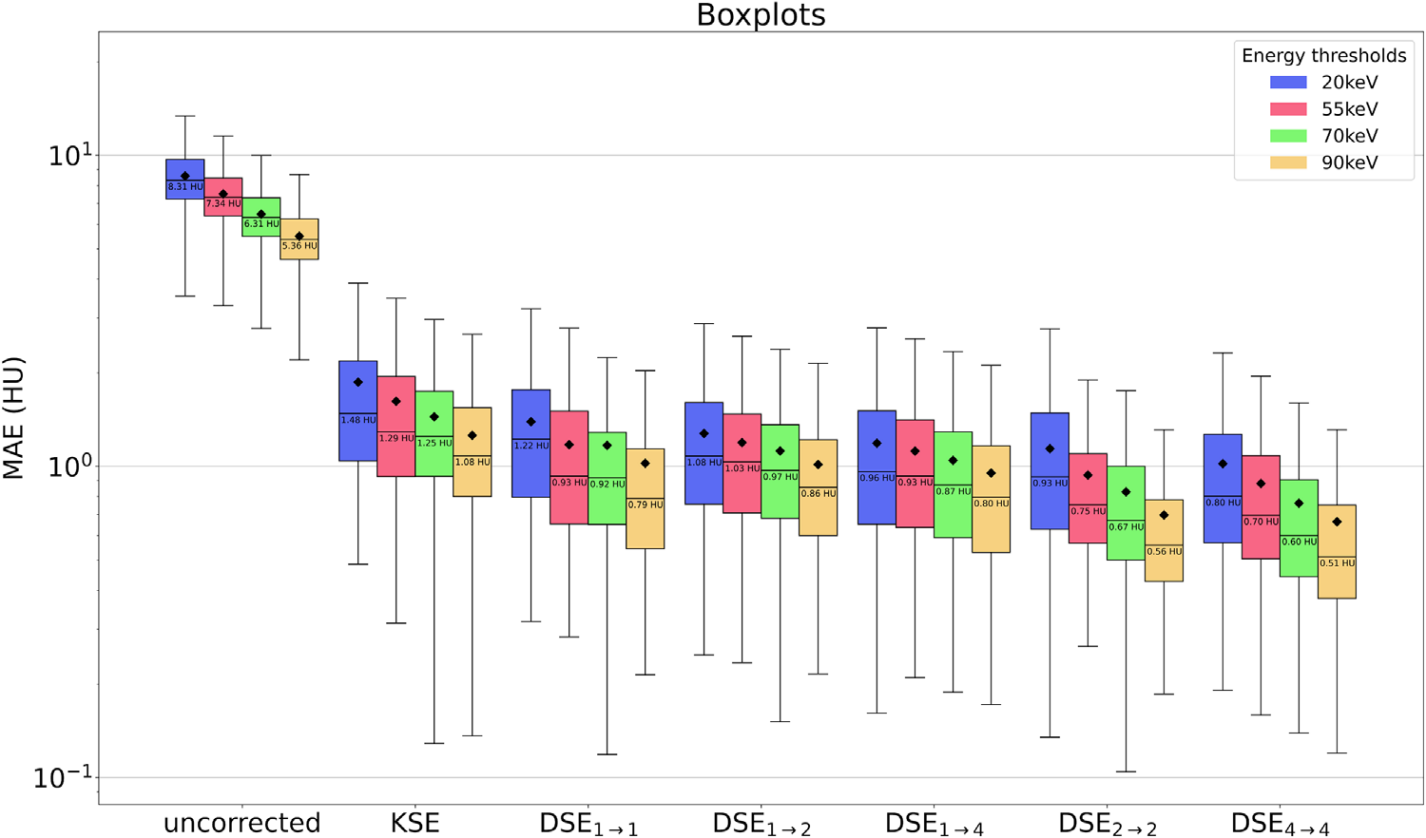
Statistical evaluation to compare the different methods for correcting scattered radiation. The different colors of the box plots indicate the different energy thresholds investigated. It should be mentioned that the *y*‐scale was chosen logarithmic so that the difference between the methods is more clearly visible.

**FIGURE 7:**
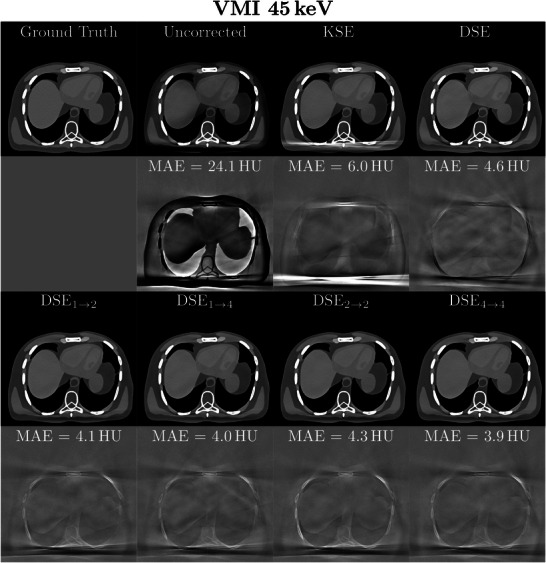
Example of patient and bowtie scatter correction for 45 keV VMIs. Seven different scatter correction methods are shown as well as the ground truth (rows 1 and 3). Each MAE excluding air is shown in the difference to ground truth images (rows 2 and 4). The reconstruction was performed with a Qr40 kernel and a 350 mm FOV. Reconstructed images: *C*=40 HU, *W*=250 HU. Difference images: *C*=0 HU, *W*=80 HU.

**FIGURE 8:**
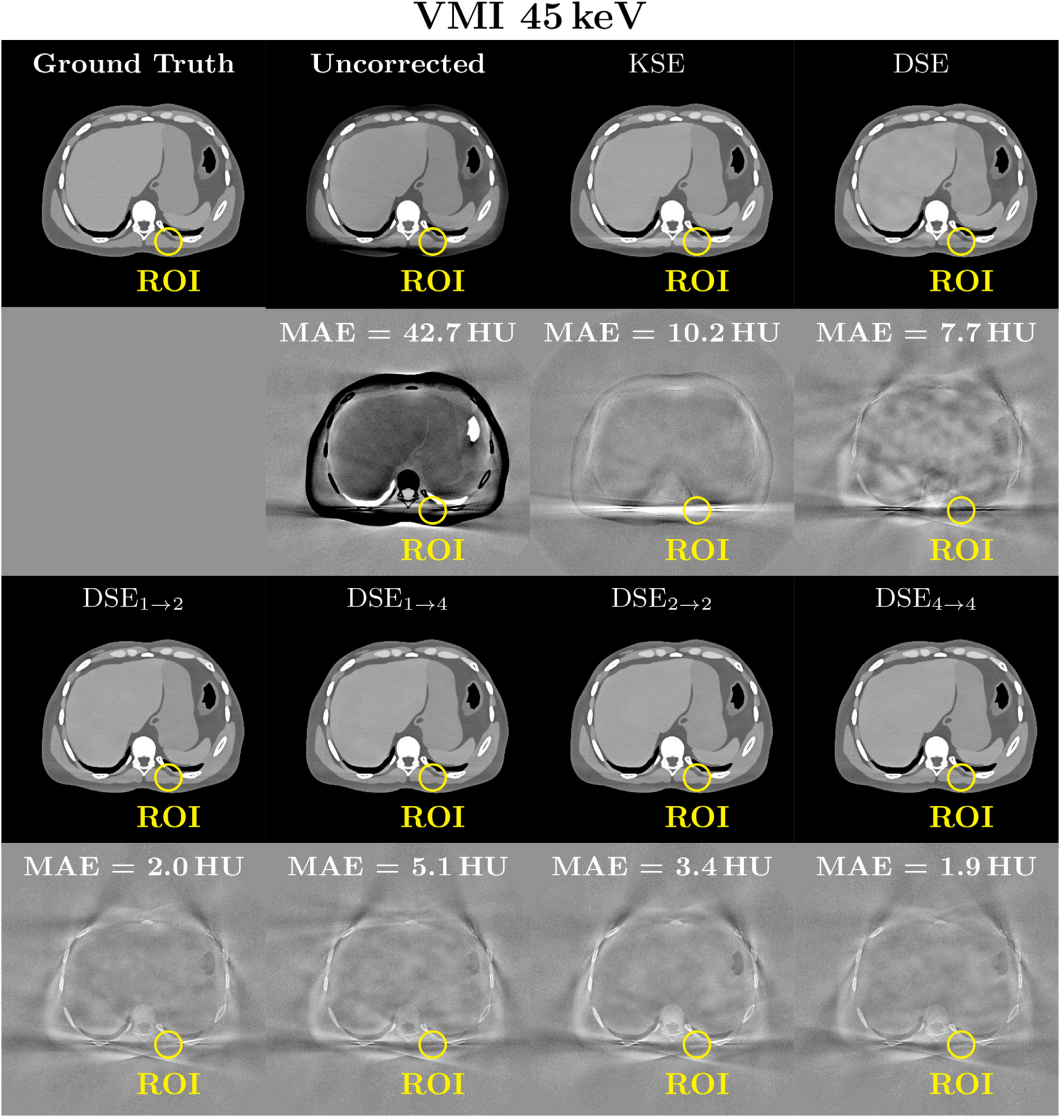
Example of patient and bowtie scatter correction for 45 keV VMIs. Seven different scatter correction methods are shown as well as the ground truth (rows 1 and 3). Each MAE for the region of interest is shown in the difference to ground truth images (rows 2 and 4). The reconstruction was performed with a Qr40 kernel and a 450 mm FOV. Reconstructed images: *C*=40 HU, *W*=250 HU. Difference images: *C*=0 HU, *W*=80 HU.

**FIGURE 9:**
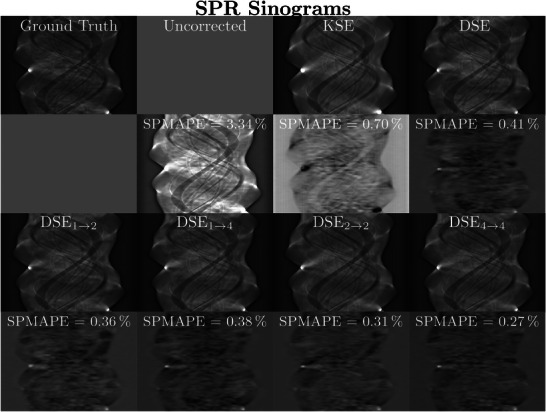
Example of SPR sinograms for the different scatter estimation methods of a simulated XCAT abdomen phantom (20 keV threshold). The scatter estimates of seven different methods divided by the primary intensity are shown as well as the MC simulated ground truth (rows 1 and 3). The SPMAPE is shown in the difference to ground truth images (rows 2 and 4). SPR sinograms: *C*=0.0, *W*=0.25. Difference SPR sinograms: *C*=0.01, *W*=0.1.

**FIGURE 10:**
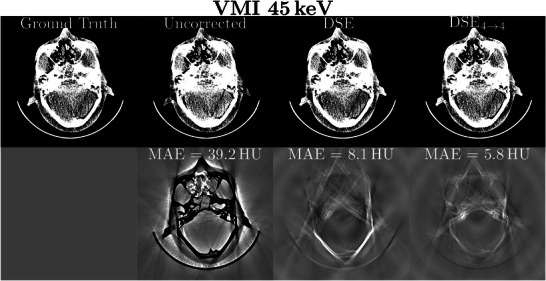
Example of patient and bowtie scatter correction for real measurements of an anthropomorphic head phantom. The best spectral DSE method as well as the non‐spectral DSE are shown. In addition, the slit scan reconstruction and the uncorrected images are displayed. Each MAE for the region of interest is shown in the difference to the slit scan images. The reconstruction was performed with a Qr40 kernel and a 280 mm FOV. Reconstructed images: *C*=40 HU, *W*=100 HU. Difference images: *C*=0 HU, *W*=100 HU.

**FIGURE 11:**
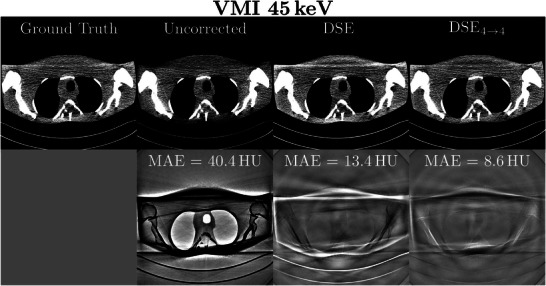
Example of patient and bowtie scatter correction for real measurements of an anthropomorphic thorax phantom. The best spectral DSE method as well as the non‐spectral DSE are shown. In addition, the slit scan reconstruction and the uncorrected images are displayed. Each MAE for the region of interest is shown in the difference to the slit scan images. The reconstruction was performed with a Qr40 kernel and a 380 mm FOV. Reconstructed images: *C*=40 HU, *W*=250 HU. Difference images: *C*=0 HU, *W*=100 HU.

### 2.2 Measured test data set

For the evaluation of our DSE networks and the reference method we obtained measurements of a head phantom (consisting of a real human skull induced with several marker objects inside the head and covered by epoxy resin) and an anthropomorphic thorax phantom at a Siemens NAEOTOM Alpha.Peak^®^ scanner. 360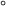 circle scans with 1008 projections per scan were performed. The measurements were done with a tube voltage of 140 kV and 200 mA tube current. The default wide bowtie filter was active. As comparison for the performance of the different approaches, a slit scan was performed with an explicit collimation of 2.4 mm in aperture plane. With this narrow collimation, it is assumed that scattered radiation can be neglected in the directly opposite detector rows. The scans were carried out in research mode, which allows up to four thresholds to be recorded. Scans were performed with the same threshold combination as in the simulation.

### 2.3 Loss function

During training, the scatter‐to‐primary‐weighted mean absolute percentage error (SPMAPE) is used as the loss function. This metric was first introduced by Erath et al.^17^ The SPMAPE accounts for the fact that an increased scatter‐to‐primary ratio directly deteriorates image quality.^17^ In regions where this ratio is high, more pronounced artifacts appear in the reconstructed images. Specifically, in areas where the primary signal is low, scatter significantly affects image quality. Therefore, accurate correction in these regions is particularly crucial. The SPMAPE has been implemented as loss function for DSE networks presented in this work as well as the kernel‐based scatter estimation (KSE), which is used as comparison method. Depending on the network architecture, the $I_{\text{scatter}}$ term represents either bowtie scatter, patient scatter, or a combination of both, while $I_{\text{pri}}$ refers to the primary intensity. Given $I_{\text{scatter, MC}}$ as the MC‐simulated scatter distribution, $I_{\text{scatter,}\;\chi }$ as the scatter intensity estimated by the correction method χ∈{DSE,KSE}, and N as the number of detector pixels, the SPMAPE is defined as follows:

(1) $$\begin{array}{cc} {\text{SPMAPE}}_{\chi } & =\frac{100\%}{N}\sum_{N}\left|\frac{I_{\text{scatter,}\;\chi }-I_{\text{scatter, MC}}}{I_{\text{scatter, MC}}}\cdot \frac{I_{\text{scatter, MC}}}{I_{\text{pri}}}\right|\\ & =\frac{100\%}{N}\sum_{N}\left|\frac{I_{\text{scatter,}\;\chi }-I_{\text{scatter, MC}}}{I_{\text{pri}}}\right|. \end{array}$$

### 2.4 DSE for patient and bowtie scatter

In recent years, it has been demonstrated that DSE can be applied to various use cases. All these studies focus on the correction of patient‐induced scattered radiation, which is the primary cause of artifacts in reconstructed images (see Section A.3). Nevertheless, other objects within the X‐ray beam path can also generate scatter and can even exceed the scatter caused by the patient, for example,  in peripheral areas of the patient. Hennemann et al.^24^ have demonstrated that DSE is capable of correcting not only for patient scatter but also for bowtie scatter. This section of the study aims to investigate whether two separate networks are necessary, one for estimating bowtie scatter and another for patient scatter, or if a single network is sufficient to estimate both scatter types simultaneously. For this purpose, the DSE network is adapted to coarse ASGs as found in PC detectors. The DSE architecture from Erath et al.^34^ has been modified accordingly to enable the simultaneous estimation of both bowtie and patient scatter.

As input, the network receives six different input channels, each of which corresponds to a different pixel position (named A–F) within the lamellae of the ASG distributed on the whole detector (see Figure 1). This is used to maintain the low frequency characteristic that scatter typically follows. Scatter intensities according to the whole detector and the different pixel positions within the ASG can be seen in Figure 2. After the scatter estimation, the six output channels of DSE are then merged according to their position within the ASG to obtain the scatter correction term for the whole detector. This splitting in and merging of the six sub‐signals is performed for all methods proposed in this paper and is visualized in Figure 4. The architecture of DSE can be seen in Figure 5. For clarity, the six input and output channels are not listed individually in the image relating to the architecture, but are abbreviated with a “6 ×” symbol. The input to the network consists of individual projections, which include not only the primary intensity but also the forward‐scattered radiation from both the patient and the bowtie filter:

(2) $$p=-\ln(I_{\text{pri}}+I_{\text{fwd}}+I_{\text{bwt}}).$$

$I_{\text{pri}}$, $I_{\text{fwd}}$, and $I_{\text{bwt}}$ are the intensities normalized by the unattenuated air intensity. $I_{\text{bwt}}$ is the intensity of the scattered radiation in the bowtie attenuated by the patient. $I_{\text{fwd}}$ is the patient scatter intensity. Two approaches were tested in this work to estimate patient and bowtie scatter. These networks were trained using data from only the lowest energy threshold (20 keV energy threshold), as the objective was solely to evaluate their performance while accounting for the influence of the bowtie filter. Both networks used the SPMAPE as loss function as introduced in Section 2.3.

#### 2.4.1 Separate estimation of bowtie and patient scatter

This approach consists of two separate networks: one for estimating patient scatter and another for bowtie scatter. Both networks have an identical architecture and use the input mapping described in (2). The patient scatter network outputs only $I_{\text{fwd}}$, while the bowtie scatter network estimates only $I_{\text{bwt}}$. Both networks have the same number of filters (filters = 24) in the first convolutional layer and, consequently, an identical number of network parameters. These values can be found in Table 2.

**TABLE 2:** Overview of the different network architectures for the estimation of patient and bowtie scatter. The names of the networks are shown on the left. The exact usage description is given on the right. The size of the input and output layers and the number of network parameters are shown in the middle.

Algorithm name	Input size	Output size	Network parameters	Description
Separately trained DSE	688 ×48× 6	688 ×48× 6	4 856 238	Networks which separately estimate patient and bowtie scatter; trained twice
Jointly trained DSE	688 ×48× 6	688 ×48× 6	8 631 724	Network which jointly estimate patient and bowtie scatter; trained once

#### 2.4.2 Joint estimation of bowtie and patient scatter

The second approach consists of a single network designed to estimate both outputs ($I_{\text{fwd}}+I_{\text{bwt}}$) simultaneously. This network has 32 filters and therefore almost as many parameters for solving the whole problem as the separately trained networks. The input of the network is defined by (2).

### 2.5 Spectral DSE

Each energy threshold contains a different proportion of scattered photons, which can be advantageous for scatter estimation and is looked into in this study. To investigate this, multiple networks are trained, differing primarily in the number of energy thresholds available for scatter estimation. The following sections provide a detailed description of the different network architectures. We compare a base model (DSE) with several spectral variants (${\mathrm{DSE}}_{x\to y}$), where x denotes the number of energy thresholds used as input and y denotes the number of energy thresholds predicted by the network. For example, ${\mathrm{DSE}}_{1\to 4}$ uses one energy threshold as input to estimate the scatter for four spectral channels, while ${\mathrm{DSE}}_{2\to 2}$ and ${\mathrm{DSE}}_{4\to 4}$ use two and four in‐ and outputs, respectively. According to this nomenclature, DSE could also be written as ${\mathrm{DSE}}_{1\to 1}$, but for the sake of simplicity this is not done. A short summary of the networks is given in Table 3. The network architectures are also based on DSE for coarse ASG^34^ but slightly modified to estimate scatter for several energy thresholds. A detailed overview of the network architectures is provided in Figure 5, in which the additional possible inputs and outputs are grayed out. The same splitting into sub‐signals is performed as mentioned before. Since the results from Section 2.4 indicate that separate networks are not required, the networks in this part of the study are trained to estimate both bowtie and patient scatter simultaneously. The input mapping follows (2), while the output consists of $I_{\text{fwd}}+I_{\text{bwt}}$.

**TABLE 3:** Overview of the different network architectures.

Algorithm	Input size	Output size	Filters in first layer	Network parameters	Description
DSE	688 × 48× 6	688 × 48×6	32	8 631 724	gets one energy threshold as input and estimates one. is trained four times
${\mathrm{DSE}}_{1\to 2}$	688 × 48 × (6 × 1)	688 × 48 × (6 × 2)	44	16 317 028	gets one energy threshold as input and estimates two. is trained two times
${\mathrm{DSE}}_{1\to 4}$	688 × 48 × (6 × 1)	688× 48 × (6 × 4)	64	34 518 128	gets one energy threshold as input and estimates four. is trained one time
${\mathrm{DSE}}_{2\to 2}$	688 × 48 × (6 × 2)	688 × 48 × (6 × 2)	44	16 316 524	gets two energy threshold as input and estimates two. is trained two times
${\mathrm{DSE}}_{4\to 4}$	688 × 48 × (6 × 4)	688 × 48 × (6 × 4)	64	34 528 496	gets four energy threshold as input and estimates four. is trained one time

*Note*: The names of the networks are shown on the left. The exact description of the use and the number of times a net has been trained can be found on the right‐hand side. The size of the input and output layers as well as the number of filters and the corresponding number network parameters are shown in the middle. For the size of the inputs/outputs, the last number in brackets indicates the number of energy thresholds used.

#### 2.5.1 DSE

As a reference method, DSE is implemented as described by Erath et al.^34^ It is designed to process a single energy threshold per training instance. To account for four different thresholds, the network is trained separately for each, using data corresponding to the respective threshold.

#### 2.5.2 DSE_1→ 2_

In a PC detector with multiple energy thresholds T, the detected photons follow a hierarchical structure: each higher threshold contains only a subset of the photons recorded at lower thresholds. Since T1 already includes all of the photon information from the higher thresholds, the information provided by T2 is, to some extent, redundant. This property can be leveraged to reduce the input data for the neural network without losing essential information for scatter correction. Based on this observation, an alternative network architecture, ${\mathrm{DSE}}_{1\to 2}$, was implemented. Instead of utilizing both T1 and T3 as input, this network estimates the scatter signal for both thresholds using only the data from T1. A second network was trained identically for thresholds T2 and T4, with T2 serving as the sole input. The number of filters was adjusted to ensure that the total number of network parameters closely matches that of DSE.

#### 2.5.3 DSE_1→ 4_

${\mathrm{DSE}}_{1\to 4}$ network was designed to estimate the scatter signal for all four thresholds (T1 to T4) using only the data from the lowest threshold (T1) as input. This approach exploits the fact that T1 inherently contains all photon information from the higher thresholds, making additional input channels potentially redundant for scatter estimation. The network architecture was adapted accordingly, with the number of filters adjusted to maintain a comparable parameter count to previous models.

#### 2.5.4 DSE_2→ 2_

${\mathrm{DSE}}_{2\to 2}$ processes two energy thresholds simultaneously to utilize spectral information for scatter correction. To achieve this, the architecture was modified to accept 12 input channels, with six channels representing each threshold. The output layer retains the same size, ensuring that the full detector signal is reconstructed by appropriately merging the six individual pixel signals per threshold. The network was trained in two separate instances: first using thresholds T1 and T3, and then with thresholds T2 and T4. To ensure a comparable model complexity, the number of filters in the first layer was adjusted that the combined parameter count of both networks is nearly identical to that of the four separately trained networks in the standard DSE.

#### 2.5.5 DSE_4→ 4_

${\mathrm{DSE}}_{4\to 4}$ shares the same overall architecture as ${\mathrm{DSE}}_{2\to 2}$, differing primarily in the number of channels in its input and output layers. Both layers consist of 24 channels, corresponding to four energy thresholds. To maintain consistency with DSE, the number of filters in the first layer was adjusted so that the total number of network parameters remains unchanged.

### 2.6 KSE

As a physics‐driven comparison, we implemented the KSE.^35^ Originally designed for one‐dimensional fan‐beam CT systems, the method has since been extended to two dimensional detector geometries. The KSE method estimates the total scatter signal $S_{\text{est}}\left(u,v\right)$ at detector position (u,v) as a spatial integration over a scatter source term $T(p\left(u^{\prime },v^{\prime }\right))$ and a propagation kernel $G(u,v,u^{\prime },v^{\prime };c)$, which models the spatial contribution of scattered photons originating at position $\left(u^{\prime },v^{\prime }\right)$ and detected at (u,v), depending on a parameter set c:

(3) $$S_{\text{est}}\left(u,v\right)=\;\int \int \;T\left(p\left(u^{\prime },v^{\prime }\right)\right)\cdot G\left(u,v,u^{\prime },v^{\prime };c\right)du^{\prime }dv^{\prime }$$

In the case of a stationary kernel, the operation simplifies to a convolution:

(4) $$S_{\text{est}}\left(u,v\right)=T\left(p\left(u,v\right)\right){*}_{u}K_{u}\left(u\right){*}_{v}K_{v}\left(v\right)$$

where the kernel is assumed to be separable, $K\left(u,v\right)=K_{u}\left(u\right)\cdot K_{v}\left(v\right)$, and the source term T(p(u,v)) is defined as:

(5) $$T\left(p\left(u,v\right)\right)=\kappa \cdot p\left(u,v\right)\cdot e^{-\gamma \cdot p(u,v)}$$

The post‐log projection p(u,v) is derived from the air‐normalized primary intensity via:

(6) $$p\left(u,v\right)=-\log\left(\frac{I(u,v)}{I_{0}\left(u,v\right)}\right)$$

To achieve reliable estimates, the free parameters κ,γ and the kernel parameters c were calibrated using MC simulation data, as described in Section 2.1. Parameter optimization was performed by minimizing the loss function in (1) using the same training data as for the proposed DSE methods. For robustness, the KSE model was optimized independently for four different energy thresholds.

### 2.7 Training details

The networks proposed in this work are implemented in Tensorflow version 2.10. The training was carried out on an NVIDIA V100 Tesla GPU. We used the Glorot uniform initialization^36^ for initializing the weights and set the bias to zero. To avoid overfitting we conducted early stopping, when the validation loss has not changed for 100 epochs. Every 25 epochs without any change in the validation loss the learning rate was reduced by a factor of two. The initial learning rate was set to $10^{-4}$. The Adam optimizer was used for training. Batch size was set to 32. Depending on the network architecture the number of convolutional filters was set individual. All networks were trained for 500 epochs. On the specified GPU hardware, single‐threshold models required approximately 6 h of computation time, whereas multi‐threshold models took up to 11 h. Specifically, the runtimes were as follows: ${\mathrm{DSE}}_{1\to 2}$ = 9 h, ${\mathrm{DSE}}_{1\to 4}$ = 11 h, ${\mathrm{DSE}}_{2\to 2}$ = 9 h and ${\mathrm{DSE}}_{4\to 4}$ = 11 h. The simulated training data set consists of 7560 projection pairs per energy threshold. For networks with a single threshold, such as DSE, the model uses 7560 projections. For architectures involving multiple thresholds as input or output, such as ${\mathrm{DSE}}_{1\to 2}$, ${\mathrm{DSE}}_{1\to 4}$, ${\mathrm{DSE}}_{2\to 2}$, or ${\mathrm{DSE}}_{4\to 4}$, the same projections are used for each threshold, which means the effective data size scales with the number of thresholds. For example, ${\mathrm{DSE}}_{1\to 2}$ processes two sets of 7560 projections, while ${\mathrm{DSE}}_{1\to 4}$ processes four sets. The corresponding filter counts and total parameters for each network can be found in Tables 2 and 3.

### 2.8 Evaluation

Our proposed methods are evaluated both in projection and in image domain. For the comparison in projection domain, the SPMAPE metric is used. Since SPMAPE directly correlates with reconstruction errors, it serves as a strong indicator of a network's performance in image domain as well. Subsequently, the spectral performance of the various DSE approaches (introduced in Section 2.5) is assessed. Initially, the evaluation is performed on a simulated test data set, considering both projection and image domains. Furthermore one‐sided paired Wilcoxon‐tests were conducted for all combinations of network predictions to assess whether one network significantly outperformed another. Simulated ground truth images were reconstructed from primary‐only projections. This ensures that reconstruction errors shown in the figures can be attributed to scatter correction performance rather than residual scatter in the reference data. The evaluation was based on a data set comprising 8496 reconstructed slices and was carried out separately for each of the four energy thresholds. This allowed for a robust comparison of network performance across spectral conditions. In our study, VMIs at 45, 70, and 100 keV were generated by applying weighted linear combinations of the threshold‐based images, independent of manufacturers implementation. We selected this method to maintain consistency across configurations with up to four energy thresholds and to avoid introducing additional complexity unrelated to the primary research objective of evaluating scatter correction. Each threshold‐based image was reconstructed from projection data that had first been corrected using the scatter‐correction methods presented in this work. Regarding scatter behavior, forming VMIs through linear combinations means that residual scatter contributions from the DSE‐corrected threshold images propagate proportionally according to the applied weights into the VMI images. Consequently, no new artifacts are introduced beyond those present in the original threshold images, although weighting can amplify or attenuate existing scatter components. In addition to specifying the global MAE over the entire reconstructed volume, we defined a critical MAE (MAE_10_) limited to voxels whose error in the uncorrected image exceeded 10 HU compared to the ground truth. A threshold of 10 HU was chosen since deviations of this magnitude in soft tissue are generally considered visually perceptible and exceed the expected noise level of modern CT systems.^37^, ^38^, ^39^ In our test data set, MAE_10_ comprised approximately 25 % of all voxels, thus providing a targeted measure of error in clinically critical areas. Finally, slit scan data, as described in Section 2.2, are used as reference for our measurements. It contains only a small amount of scatter compared to the full cone‐beam scan. The same scan with full collimation is corrected using the proposed method. In order to evaluate the performance of all the proposed methods, the scatter‐corrected projections are reconstructed with the filtered back projection algorithm. All reconstructions in this study were performed using the Qr40 and Qr60 kernels from the Siemens quantitative regular kernel family. These kernels are specifically designed for imaging applications where accurate CT values are essential. The numeric value indicates the relative sharpness: Qr40 offers a balanced edge definition suitable for soft tissue, while Qr60 applies stronger high‐frequency filtering to enhance detail resolution, typically used for bone or other high‐contrast structures. Although any kernel can be used for image reconstruction and the choice of kernel is independent of the correction process, this work focuses on these two kernels for illustration and comparison purposes.

## 3 RESULTS

### 3.1 Comparison between simultaneous and separated bowtie and patient scatter estimation

First, DSE with separately trained networks for patient and bowtie scatter and DSE with jointly scatter estimation for both scatter types are compared. All other parameters such as learning rate, batch size, network parameters etc. were kept identical. Table 4 shows the results of the scatter correction for the simulated testing data set. The global MAE of 8.3 HU is reduced to 1.3 HU by the separately trained DSE. The ability to estimate both scatter types combined leads to a reduced MAE of 1.4 HU. Even in regions where artifacts are more visible, both methods perform equally well (see MAE_10_). The reference method KSE is able to reduce the MAE to 1.9 HU. Regarding the maximum MAE and the SPMAPE in the reconstructed test data set both DSE networks perform almost identically. Both networks outperform the conventional KSE. Section A.3 shows a detailed overview of the distribution of the individual scatter types in relation to the total scatter signal.

**TABLE 4:** Results of the correction of bowtie and patient scatter separately or jointly. The methods were evaluated using the lowest energy threshold (20 keV).

Correction method	MAE (HU)	MAE_10_ (HU)	Min./Max. MAE (HU)	SPMAPE
Uncorrected	8.3 ± 2.3	23.8 ± 7.4	0.4/29.0	1.41 ± 0.62
KSE	1.9 ± 1.3	3.5 ± 2.6	0.5/11.5	0.31 ± 0.18
Separately trained DSE	1.3 ± 0.8	2.2 ± 1.35	0.1/4.6	0.21 ± 0.13
Jointly trained DSE	1.4 ± 0.8	2.4 ± 2.3	0.3/4.5	0.21 ± 0.12

### 3.2 Spectral scatter correction

Table 5 compares the different network performances at four different energy thresholds. It can be observed that DSE is able to clearly reduce the image MAE for all four energy thresholds. Nevertheless, all networks that incorporate the spectral information inherent in the PCCT scan for scatter correction outperform DSE significantly (except for ${\mathrm{DSE}}_{1\to 2}$, which performs worse than DSE for the three higher energy thresholds). With these additional inputs the network is able to leverage information from different energy threshold levels for scatter estimation. From the proposed network architectures ${\mathrm{DSE}}_{4\to 4}$ leads to the best results and shows the best SPMAPE and MAE in the scatter‐corrected images for all energy thresholds. It significantly outperforms all other tested networks for all four energy thresholds. ${\mathrm{DSE}}_{2\to 2}$, ${\mathrm{DSE}}_{1\to 2}$, and ${\mathrm{DSE}}_{1\to 4}$ achieve comparable results for the SPMAPE and MAE in the reconstructed images and outperform DSE. A significant difference for all energy thresholds, however, can only be observed for ${\mathrm{DSE}}_{2\to 2}$ and ${\mathrm{DSE}}_{1\to 4}$. When comparing the remaining maximum error in the corrected images the spectral methods are the most robust, closely followed by DSE with slightly worse results for the two higher thresholds. All the proposed networks are compared with KSE. The proposed networks all show significantly better results than the comparison method over all energy levels. For a better presentation of the quantitative results, they were visualized as a boxplot (Figure 6). This illustrates once more that ${\mathrm{DSE}}_{4\to 4}$ delivers the best results for the complete test data set. It is also clear that all deep learning methods outperform the conventional approach. The significance test results are shown in Section A.2. The advantage of spectral methods is even more apparent with VMIs. Especially for low keV VMIs, where the lower thresholds are weighted more heavily, spectral networks outperform the non spectral DSE (Table 6). Figure 7 shows an example of the scatter correction for a 45 keV VMI. In reconstructed images, the forward scatter artifacts are almost completely corrected with the proposed neural networks. Only in the difference to the ground truth images some artifacts still remain visible. The KSE method is clearly over‐correcting in some areas, where the DSE method is under‐correcting. For the proposed spectral DSE methods the main difference is seen in bone areas, where all networks seem to over‐correct. It can also be seen that the results are superior to KSE. In Figure 8 another example for the scatter correction is shown. Without scatter correction the MAE in the region of interest (ROI) is 42.7 HU. The KSE reduces the error to 10.2 HU, while DSE leads to an MAE of 7.7 HU. The best correction is achieved with ${\mathrm{DSE}}_{4\to 4}$ (1.9 HU).

**TABLE 5:** Quantitative results for four energy thresholds (20, 55, 70, and 90 keV), evaluated for patient data and for 45 FORBILD head phantoms. A total of 8496 slices with a thickness of 0.4 mm were analyzed. Reconstructions were done with a 500 mm FOV and Qr60 kernel.

Correction method	MAE (HU)	MAE_10_ (HU)	Min./Max. MAE (HU)	SPMAPE
**20 keV energy threshold**				
Uncorrected	8.6 ± 2.3	23.8 ± 7.4	0.4/29.0	1.57 ± 0.70
KSE	1.9 ± 1.3	3.5 ± 2.6	0.5/11.5	0.31 ± 0.18
DSE	1.4 ± 0.8	2.4 ± 2.3	0.3/4.5	0.21 ± 0.12
${\mathrm{DSE}}_{1\to 2}$	1.3 ± 0.7	1.8 ± 1.2	0.3/4.3	0.21 ± 0.11
${\mathrm{DSE}}_{1\to 4}$	1.2 ± 0.7	1.8 ± 1.2	0.2/4.5	0.20 ± 0.12
${\mathrm{DSE}}_{2\to 2}$	1.1 ± 0.7	2.0 ± 1.4	0.1/4.2	0.19 ± 0.11
${\mathrm{DSE}}_{4\to 4}$	1.0 ± 0.7	1.6 ± 1.1	0.2/4.1	0.19 ± 0.11
**55 keV energy threshold**				
Uncorrected	7.5 ± 2.0	22.8 ± 6.2	0.2/25.0	1.34 ± 0.57
KSE	1.6 ± 0.7	3.2 ± 2.4	0.3/10.0	0.27 ± 0.16
DSE	1.2 ± 0.7	1.7 ± 1.3	0.3/4.1	0.20 ± 0.14
${\mathrm{DSE}}_{1\to 2}$	1.2 ± 0.7	2.2 ± 1.8	0.2/3.6	0.18 ± 0.10
${\mathrm{DSE}}_{1\to 4}$	1.1 ± 0.7	1.8 ± 1.2	0.2/3.8	0.18 ± 0.12
${\mathrm{DSE}}_{2\to 2}$	0.9 ± 0.6	1.7 ± 1.2	0.3/3.8	0.16 ± 0.09
${\mathrm{DSE}}_{4\to 4}$	0.9 ± 0.6	1.5 ± 1.0	0.2/3.7	0.16 ± 0.09
**70 keV energy threshold**				
Uncorrected	6.5 ± 1.8	21.2 ± 4.9	0.1/22.0	1.12 ± 0.48
KSE	1.4 ± 0.6	3.2 ± 2.2	0.1/8.4	0.23 ± 0.13
DSE	1.2 ± 1.0	1.8 ± 1.1	0.1/7.7	0.19 ± 0.18
${\mathrm{DSE}}_{1\to 2}$	1.1 ± 0.6	1.7 ± 1.3	0.2/3.9	0.17 ± 0.10
${\mathrm{DSE}}_{1\to 4}$	1.0 ± 0.6	1.8 ± 1.3	0.2/3.9	0.17 ± 0.11
${\mathrm{DSE}}_{2\to 2}$	0.8 ± 0.5	1.6 ± 1.0	0.1/3.7	0.14 ± 0.08
${\mathrm{DSE}}_{4\to 4}$	0.8 ± 0.5	1.3 ± 0.8	0.1/3.8	0.14 ± 0.08
**90 keV energy threshold**				
Uncorrected	5.5 ± 1.5	19.5 ± 4.1	0.3/19.0	0.92 ± 0.39
KSE	1.3 ± 0.7	3.0 ± 2.1	0.1/7.0	0.19 ± 0.12
DSE	1.0 ± 0.9	1.5 ± 0.9	0.2/6.5	0.17 ± 0.16
${\mathrm{DSE}}_{1\to 2}$	1.0 ± 0.6	2.1 ± 1.9	0.2/3.9	0.15 ± 0.08
${\mathrm{DSE}}_{1\to 4}$	1.0 ± 0.6	1.7 ± 1.3	0.2/3.9	0.15 ± 0.09
${\mathrm{DSE}}_{2\to 2}$	0.7 ± 0.5	1.3 ± 0.8	0.2/3.8	0.12 ± 0.07
${\mathrm{DSE}}_{4\to 4}$	0.7 ± 0.5	1.1 ± 0.8	0.1/3.8	0.11 ± 0.07

**TABLE 6:** Quantitative results for VMIs at three different energy levels (45, 70, and 100 keV), evaluated for patient data and for 45 FORBILD head phantoms. A total of 8496 slices with a thickness of 0.4 mm were analyzed. Reconstructions were done with a 500 mm FOV and Qr60 kernel.

Correction method	MAE (HU)	MAE_10_ (HU)	Min./Max. MAE (HU)
**45 keV VMI**			
Uncorrected	16.4 ± 5.0	26.1 ± 8.3	2.5/53.3
KSE	4.3 ± 2.8	5.4 ± 3.8	1.6/24.8
DSE	3.5 ± 1.7	4.1 ± 2.6	1.3/9.9
${\mathrm{DSE}}_{1\to 2}$	2.6 ± 1.3	3.0 ± 1.6	1.2/10.5
${\mathrm{DSE}}_{1\to 4}$	2.6 ± 1.4	3.0 ± 1.7	1.1/11.2
${\mathrm{DSE}}_{2\to 2}$	2.6 ± 1.4	3.0 ± 1.8	1.1/10.5
${\mathrm{DSE}}_{4\to 4}$	2.4 ± 1.2	2.7 ± 1.5	0.9/9.6
**70 keV VMI**			
Uncorrected	8.0 ± 2.2	22.4 ± 6.9	0.3/27.2
KSE	1.7 ± 1.7	3.3 ± 2.4	0.4/10.8
DSE	1.1 ± 1.0	1.8 ± 1.3	0.4/10.8
${\mathrm{DSE}}_{1\to 2}$	1.1 ± 0.5	1.7 ± 1.2	0.2/3.8
${\mathrm{DSE}}_{1\to 4}$	1.0 ± 0.6	1.8 ± 1.2	0.2/4.2
${\mathrm{DSE}}_{2\to 2}$	0.9 ± 0.5	1.7 ± 1.1	0.2/3.6
${\mathrm{DSE}}_{4\to 4}$	0.8 ± 0.5	1.5 ± 0.9	0.2/3.0
**90 keV VMI**			
Uncorrected	5.4 ± 1.5	18.9 ± 4.1	0.4/18.4
KSE	1.3 ± 0.5	3.0 ± 2.0	0.3/6.7
DSE	0.9 ± 0.4	1.8 ± 1.1	0.3/3.2
${\mathrm{DSE}}_{1\to 2}$	0.9 ± 0.4	1.6 ± 1.3	0.3/3.4
${\mathrm{DSE}}_{1\to 4}$	0.9 ± 0.5	1.7 ± 1.4	0.2/3.8
${\mathrm{DSE}}_{2\to 2}$	0.6 ± 0.3	1.3 ± 0.7	0.3/2.3
${\mathrm{DSE}}_{4\to 4}$	0.6 ± 0.3	1.2 ± 0.8	0.2/2.3

In addition to the image‐domain evaluation, we also analyze the performance of the proposed methods in the projection domain. Figure 9 presents scatter‐to‐primary sinograms for a simulated abdominal phantom, as the SPR is known to correlate strongly with reconstruction errors. We show both the estimated scatter divided by the primary intensity as well as the corresponding difference to the Monte Carlo–based ground truth. For quantitative evaluation, we provide the SPMAPE metric, as it correlates directly with reconstruction errors and thus represents a meaningful measure of the accuracy of the scatter estimation. Across all projection views, ${\mathrm{DSE}}_{4\to 4}$ achieves the lowest SPMAPE and shows the smallest errors in the SPR sinograms, indicating the most accurate scatter estimation. ${\mathrm{DSE}}_{2\to 2}$ performs similarly well, with SPMAPE values close to those of ${\mathrm{DSE}}_{4\to 4}$ and comparably low deviations from the ground truth. In contrast, DSE, ${\mathrm{DSE}}_{1\to 2}$, and ${\mathrm{DSE}}_{1\to 4}$ exhibit slightly higher SPMAPE values. KSE performs substantially worse than all DSE variants, resulting in pronounced under‐ or overestimation of scatter, particularly in regions with higher SPR. The improved consistency of ${\mathrm{DSE}}_{4\to 4}$ in the projection domain aligns with its superior reconstruction quality in the image domain.

The performance of the DSE and the best spectral DSE method (${\mathrm{DSE}}_{4\to 4}$) were evaluated for CT scans of an anthropomorphic head and an anthropomorphic thorax phantom scanned with a clinical CT scanner. To quantify the accuracy of the scatter‐corrected CT reconstructions, a slit scan measurement was performed as an example of low scatter (used as GT). The corresponding CT images and the differences to the slit scan are shown in Figures 10 and 11. As observed in the simulation study, spectral and non‐spectral DSE deliver images that are nearly free of scatter artifacts, while in the difference image of the uncorrected case, the artifacts are clearly visible. The evaluation of CT value accuracy for the head phantom shows an average deviation of 39.2 HU (uncorrected), 8.1 HU (DSE), and 5.8 HU (${\mathrm{DSE}}_{4\to 4}$) for VMI 45 keV compared to the slit scan. For the thorax phantom, the corresponding deviations are 40.4 HU (uncorrected), 13.4 HU (DSE), and 8.6 HU (${\mathrm{DSE}}_{4\to 4}$).

## 4 DISCUSSION

In addition to the well known patient scatter, scatter from the bowtie can also lead to image artifacts. To correct for both scatter types, we proposed two different deep learning‐based approaches, based on the well known DSE.

The first approach employs two separate networks, individually trained for patient and bowtie scatter estimation. This method yields very good results in correcting for both scatter types and effectively reduces residual errors in both the image and projection domains. The second approach, in contrast, simultaneously estimates both scatter types. Although its performance is only marginally lower than that of the two‐network approach, it provides the substantial advantage of requiring only a single network, thereby reducing training and deployment complexity. An aspect not considered in this study is the use of different bowtie filters in CT systems, which may introduce various artifacts. Nonetheless, given that DSE has demonstrated strong robustness to variations in scan parameters, it is reasonable to expect similarly good performance provided that appropriate training data are included^15^, ^16^.

We have introduced a novel method for deep learning‐based scatter estimation, spectral DSE, to address scatter in PCCT by utilizing the spectral information of the incident photons. We trained different neural networks that incorporate the data of several energy thresholds for scatter estimation. The methods mainly differ in the number of energy thresholds available for the correction task. Overall, non‐spectral DSE already performs very well for all energy thresholds, with only minor differences compared to the evaluated spectral approaches. Nevertheless, a consistent trend can be observed that suggests that using multiple thresholds as input and output offers an advantage (${\mathrm{DSE}}_{2\to 2}$ and ${\mathrm{DSE}}_{4\to 4}$). This effect is particularly relevant in spectral applications, where combinations of multiple thresholds are used. While the differences may seem small when considering individual thresholds, the advantage becomes apparent in spectral applications, as cumulative residual errors can be reduced more significantly, leading to a more substantial overall improvement. At the same time, the concept offers a notable practical advantage: only a single network is required, instead of training and maintaining four separate networks. This reduction in network complexity not only lowers computing and storage requirements, but can also facilitate clinical use. Furthermore, it is fundamentally unlikely that high‐frequency residual errors remaining after scatter correction will generate lesion‐like structures or obscure true pathological features. Since our method performs scatter correction directly in projection (intensity) domain rather than in image domain, it only has access to individual projections and not to the full sinogram context. Consequently, any residual error manifests as localized, rapidly oscillating deviations within individual projections that lack the spatial coherence necessary to propagate into lesion‐like patterns during reconstruction. The probability that these uncorrelated high‐frequency errors will be randomly aligned across many projection angles to form a focal, anatomically plausible artifact is extremely low, similarly low as pure projection noise would make up such lesions. Therefore, while such residual errors may contribute marginally to noise, they fundamentally lack the correlated behavior necessary to create or obscure diagnostically relevant lesions. When residuals are combined according to the ASG geometry, higher‐frequency ring‐like patterns can appear in reconstructed images, particularly when very sharp kernels are used. However, these rings differ markedly in frequency and morphology from small lesions, reducing the risk of misinterpretation. While these artifacts do not mimic lesions, we acknowledge that under‐ or overcorrection of scatter could theoretically alter local contrast and potentially obscure subtle findings. In our experiments, we did not observe such cases. To further assess generalization, we tested the method on anatomically complex phantoms and additional data sets not included in training, such as XCAT phantoms, head phantoms and scanned anthropomorphic phantoms. These results indicate robust performance beyond the original training conditions.

With an inference time of about 1.8 ms per projection, the networks are usable in the clinical workflow. Our experiments have shown that with the neural network structure and size we used, it is slightly more efficient to use a network with four thresholds to estimate forward scatter.

This study has some limitations. In case of the bowtie scatter, also other bowtie geometries should be considered, as there are different filters for different applications, for example, cardiac or pediatric imaging. Another limitation is that the results presented here only apply to forward scatter. A logical next step will be to extend the spectral methods to the estimation of cross scatter as it can occur in dual‐source devices. However, this will require an adjustment of the simulated data, a new training and adjustments in the network design.

## 5 CONCLUSIONS

In this work, we have shown that DSE can jointly estimate both bowtie and patient scatter, eliminating the need for separate networks and thereby reducing computational cost and complexity. Furthermore, we demonstrated that leveraging the spectral information of incoming photons in PCCT scans further improves performance in reducing scatter artifacts. We presented different DSE approaches that utilize energy threshold information for forward scatter correction in PCCT. All proposed spectral neural networks achieved improved results compared to conventional DSE, which cannot incorporate spectral information. Moreover, combining multiple thresholds as network input provided an additional advantage in correction. In general, scatter estimation was more accurate when more than one energy threshold was used. However, the performance gains in non‐spectral applications were limited, making the primary advantage of spectral networks their ability to replace several separate networks with a single one. In spectral applications such as VMIs, the benefit of multiple energy thresholds was more pronounced, as their combination can help mitigate residual errors.

## CONFLICT OF INTEREST STATEMENT

Lukas Hennemann, Julien Erath, Andreas Heinkele, Eric Fournié, Martin Petersilka, Karl Stierstorfer are employed by Siemens Healthineers AG.

## Figures and Tables

**FIGURE A2:**
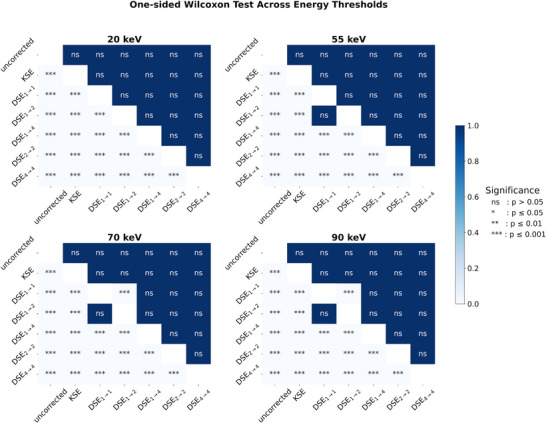
Statistical comparison of the individual methods across different energy thresholds. The matrix indicates whether method A (*y*‐axis) performs significantly better than method B (*x*‐axis) on the test data set. Statistical significance was assessed using a one‐sided paired Wilcoxon signed‐rank test. Significance levels are indicated in the legend on the right side.

## APPENDIX A

### A.1 Scatter‐to‐primary ratio analysis

In addition to scatter originating exclusively in the bowtie filter or the patient, a small fraction of detected photons undergo multiple scatter events in both objects. These contributions are fully accounted for in our MC simulation, and would lead to an increase of about 0.4 % (roughly 0.1 HU) in the MAE in the reconstructed test dataset images. Figure A1 illustrates the SPR for a 30 cm water phantom, decomposed into three components: scatter originating only in the bowtie filter (green), scatter originating only in the patient (orange), and scatter involving both the bowtie and the patient (pink). As shown, the third component is approximately two orders of magnitude smaller than the other two contributions. Since the SPR correlates directly with reconstruction error,^17^ and the MAE only increases by 0.4 %, this minor component was neglected in our data set and therefore as network targets without introducing a relevant bias. Importantly, scatter of second or higher order that occurs exclusively in the bowtie or exclusively in the patient is fully considered in our MC simulation and is included in the data set.

### A.2 One‐sided paired wilcoxon signed‐rank test

Figure A2 illustrates the significance between two different correction methods for each of the four energy thresholds. A paired one‐sided Wilcoxon signed‐rank test was applied independently at each of the reconstructions to evaluate whether values from one method were significantly better than those of the other. The test was conducted with a significance threshold of p=0.05 and a sample size of n=8496 paired observations. Methods showing statistically significant differences are highlighted in the heat map. The corresponding mapping can be seen in the legend. The results show that the ${\mathrm{DSE}}_{4\to 4}$ method clearly outperforms all other methods across all energy thresholds. Also all of the spectral methods in this work outperform KSE as well as the non‐spectral DSE. Only at higher energy thresholds does DSE perform better than the spectral methods that use only one threshold as input (${\mathrm{DSE}}_{1\to 2}$ and ${\mathrm{DSE}}_{1\to 4}$).

### A.3 Distribution of patient and bowtie scatter

This section analyzes the relative contributions of bowtie and patient scatter to the total scatter signal. To this end, MC simulations were performed in which one of the two scatter components was selectively set to zero. The resulting MAE was evaluated in reconstructions obtained with a Qr60 kernel. In total, 8496 slices from 45 FORBILD head phantoms were included in the analysis. The results are summarized in Table A1.

**TABLE A1:** Bowtie and patient scatter distribution for four energy thresholds (20, 55, 70, and 90 keV), evaluated for 45 FORBILD head phantoms and patient data. A total of 8496 slices with a thickness of 0.4 mm were analyzed. Reconstructions were done with a 500 mm FOV and Qr60 kernel.

Scatter type	MAE (HU)	Min./Max. MAE (HU)	SPMAPE
**20 keV energy threshold**			
Bowtie	3.9 ± 1.7	1.1/14.1	0.16 ± 0.08
Patient	6.8 ± 1.8	1.8/22.2	1.27 ± 0.56
**Total**	8.6 ± 2.3	0.4/29.0	1.57 ± 0.70
**55 keV energy threshold**			
Bowtie	3.4 ± 1.4	0.4/12.2	0.15 ± 0.07
Patient	6.0 ± 1.5	0.7/19.4	1.12 ± 0.47
**Total**	7.5 ± 2.0	0.2/25.0	1.34 ± 0.57
**70 keV energy threshold**			
Bowtie	2.8 ± 1.1	0.7/9.9	0.13 ± 0.05
Patient	5.4 ± 1.3	0.6/17.2	0.98 ± 0.40
**Total**	6.5 ± 1.8	0.1/22.0	1.12 ± 0.48
**90 keV energy threshold**			
Bowtie	2.3 ± 0.9	0.8/8.0	0.12 ± 0.04
Patient	4.8 ± 1.2	0.5/15.1	0.87 ± 0.34
**Total**	5.5 ± 1.5	0.3/19.0	0.92 ± 0.39
